# WT1 Promotes Cell Proliferation in Non-Small Cell Lung Cancer Cell Lines through Up-Regulating Cyclin D1 and p-pRb In Vitro and In Vivo

**DOI:** 10.1371/journal.pone.0068837

**Published:** 2013-08-01

**Authors:** Caihua Xu, Chen Wu, Yang Xia, Zhaopeng Zhong, Xiang Liu, Jing Xu, Fei Cui, Bin Chen, Oluf Dimitri Røe, Aihong Li, Yijiang Chen

**Affiliations:** 1 Department of Cardiovascular Surgery, The First Affiliated Hospital of Soochow University, Soochow, Jiangsu, China; 2 Department of Thoracic and Cardiovascular Surgery, The First Affiliated Hospital of Nanjing Medical University, Nanjing, Jiangsu, China; 3 Department of Thoracic and Cardiovascular Surgery, The First Affiliated Hospital of Guangzhou Medical University, Guangzhou, Guangdong, China; 4 Department of Thoracic and Cardiovascular Surgery, Wuxi NO.2 People’s Hospital, Wuxi, Jiangsu, China; 5 Department of Cancer Research and Molecular Medicine (IKM), Faculty of Medicine, Norwegian University of Science and Technology (NTNU), Trondheim, Norway; 6 Department of Medical Biosciences, Clinic chemistry, Umea University, Umea, Sweden; H. Lee Moffitt Cancer Center & Research Institute, United States of America

## Abstract

The Wilms’ tumor suppressor gene (WT1) has been identified as an oncogene in many malignant diseases such as leukaemia, breast cancer, mesothelioma and lung cancer. However, the role of WT1 in non-small-cell lung cancer (NSCLC) carcinogenesis remains unclear. In this study, we compared WT1 mRNA levels in NSCLC tissues with paired corresponding adjacent tissues and identified significantly higher expression in NSCLC specimens. Cell proliferation of three NSCLC cell lines positively correlated with WT1 expression; moreover, these associations were identified in both cell lines and a xenograft mouse model. Furthermore, we demonstrated that up-regulation of Cyclin D1 and the phosphorylated retinoblastoma protein (p-pRb) was mechanistically related to WT1 accelerating cells to S-phase. In conclusion, our findings demonstrated that WT1 is an oncogene and promotes NSCLC cell proliferation by up-regulating Cyclin D1 and p-pRb expression.

## Introduction

Lung cancer continues to be a major public health problem in both men and women, it is currently the cancer type with the highest mortality worldwide. The incidence has increased rapidly due to extensive tobacco smoking [1]–[3], and in China there has been a 26.9% increase in men and 38.4% in women over the past five years [4]. Non-small cell lung cancer (NSCLC) includes several histological subgroups, adenocarcinoma, squamous cell and large cell carcinoma, that comprise 80–85% of the total incidence, whereas the remaining cases include the more distinct group of small-cell lung cancer (SCLC) [2], [5]–[7]. In this study, we focus on the role of WT1 in the development and carcinogenesis of NSCLC.

The Wilms’ tumor gene (WT1) which is located at 11p13q, encodes a 52–54 kDa protein that containing four zinc finger transcriptional factors and was first identified as a tumor suppressor gene in nephroblastoma or Wilms’ tumor, a pediatric kidney cancer [8], [9]. Overexpression of this gene was also discovered in several leukemias and solid tumours, as breast cancer, lung cancer and mesothelioma, and it was hypothesized that this gene plays an oncogenic role [10], [11]. Oji Y et al suggested that WT1 plays an important role in the growth of normal lung cells; overexpression of WT1 disturb the growth and differentiation of normal lung cells and, according to their findings, lead to lung cancer [11]. WT1 has been demonstrated to play a role in the regulation of cell proliferation and apoptosis in many biological and pathological mechanisms. Recently, it has been investigated as a potential target of immunotherapy for several cancer types, including NSCLC and mesothelioma [12].

Signal transducers and activators of transcription 3 (STAT3) have been reported to be overexpressed in many human malignancies and activated by various cytokines and growth factors during cancer development and progression [13], [14]. It has been demonstrated that STAT3 promotes cancer cell proliferation via up-regulation of genes encoding apoptosis inhibitors, such as Mcl-1 and Bcl-xL and cell-cycle regulators including the cyclins D1/D2 and c-Myc [13]–[17]. Interestingly Rong et al demonstrated evidence that WT1enhanced the transcriptional activity of phosphorylated STAT3 (p-STAT3) leading to synergistic up-regulation of downstream genes including cyclin D1 and Bcl-xL, in mouse fibroblasts, melanoma and hepatic cells as well as human embryonic kidney cells [18]. However, WT1 has not been previously reported in lung cancer cell lines.

In this study, we aimed to identify the expression of WT1 protein in NSCLC specimens compared to adjacent tissues, investigate the proliferation promoting function of WT1 in vitro and in vivo and identify its relationship with p-STAT3 transcriptional activation.

## Materials and Methods

### Patients

NSCLC and corresponding adjacent tissues included in this study were obtained from 85 consecutive patients who had de novo disease and undergone surgical resection. They were included between December 2010 and April 2011 at the First Affiliated Hospital of Nanjing Medical University (Nanjing, China). The correct diagnosis was assessed by an experienced pathologist and the staging of NSCLC by a clinical oncologist according to the International Association for the Study of Lung Cancer (IASLC) 7th TNM-classification. Adjacent tissue was located within 3 cm of the edge of the tumor tissue.

### RT-PCR

RNA was obtained from snap-frozen tissues and NSCLC cell lines using Trizol (Invitrogen, Carlsbad, CA, USA) method following the manufacture’s protocol. RNA concentrations and qualities were examined by Beckman Coulter DU800 spectrophotometer (Beckman, Brea, CA, USA). cDNA were synthesized with a Primescript™ RT reagent kit (TaKaRa, Japan). 12 µL of total RNA mixed with 8 µL Primescript buffer and 20 µL DEPC-treated water was incubated at 37°C for 15 min, 85°C for 5 s and stored at 4°C until use.

### qRT-PCR

ABI Prism7500 Sequence Detector System (ABI, USA) was employed to determine the relative level of mRNA in tumor tissues and adjacent tissues. Quantitative real-time polymerase chain reaction (qRT-PCR) analysis for WT1 and β-actin was performed with SYBR® Premix ExTaq™ (TaKaRa, Japan) according to the manufacturer’s instructions. PCR was performed using 10 µl 2×Premix buffer, 0.5 µl of each 5′ and 3′ primer, and 1 µl samples or distilled water to a final volume of 20 µl. Each vial was denatured at 95°C for 1 min. denatured at 95°C for 15 sec, annealed at 60°C for 15 sec and extended at 72°C for 30 sec using the following primers: WT1 forward primer, 5′GCTATTCGCAATCAGGGTTACAG3′; WT1 reverse primer, 5′TGGGATCCTCATGCTTGAATG3′. β-actin forward primer,5′CCCAGCACAATGAAGATCAAGATCAT3′; β-actin reverse primer: 5′ATCTGCTGGAAGGTGGACAGCGA3′; at the end of the extension phase, fluorescence detection was performed. To discriminate specific from nonspecific cDNA products, a melting curve was obtained at the end of each run.

### Cell Culture

A549, H1299, H1650 and 293T cell lines (ATCC) were employed for the present study. A549, H1299 and H1650 were cultured in RPMI 1640 medium supplemented with 10% fetal bovine serum (Invitrogen, Carlsbad, CA, USA), while 293T were cultured in DMEM high glucose medium supplemented with 10% fetal bovine serum. All cells were maintained in a humidified 37°C incubator with 5% CO_2_.

### Lentivirus Production and Transduction

WT1A (-17aa-KTS isoform) gene was synthesized (purchased from Genscript, Piscataway, NJ) with restrictive digestion using Mlu I and subcloned pLV-GFP plasmid (gift from D. Beicheng Sun, University of Nanjing Medical University, China), and named pLV-GFP-WT1. To generate plasmid expressing WT1-shRNA, double-stranded oligonucleotides were cloned into pLL3.7 vector (gift from D. Yun Chen, University of Nanjing Medical University, China) and named pLL3.7-WT1-shRNA. The sequences of WT1-shRNA used are aac TCAGGGTTACAGCACGGTC ttcaagaga GACCGTGCTGTAACCCTGA tttttt c. The uppercase letters represent WT1 specific sequence and lowercase letters represent hairpin sequences. Recombinant lentivirus was generated from 293T cells using calcium phosphate precipitation. A549, H1299, H1650 were transfected with lentivirus using polybrene (8 ug/ml). Representative pictures of wild-type and transfected cells are shown in Figure S1.

### Western-blotting Assay

Proteins were extracted from cultured cells and mice tissues, quantitated using a protein assay (BCA method, Beyotime, China). Proteins were fractionated by SDS-PAGE, transferred to PVDF membrane, blocked in 4% dry milk at room temperature for 1 hour and immunostained with primary antibodies at 4°C overnight using anti-WT1 (1∶1000, 6F-H2, Millipore, USA), anti-STAT3/p-STAT3 (1∶2000, Cell Signalling Technology, Beverly, MA, USA), anti-Cyclin D1 (1∶500, Santa Cruz Biotechnology, Delaware Avenue, CA, USA), anti-p-pRb (1∶1000, Cell Signaling Technology, Beverly, MA, USA), anti-caspase3 (1∶3000, Abcam, Cambridge, MA, USA), anti-Bcl-2L (1∶1000, Abcam, Cambridge, MA, USA) and anti-GAPDH (1∶1000, Kangchen, China). The results were visualized via a chemiluminescent detection system (Pierce ECL Substrate Western blot detection system, Thermo, Pittsburgh, PA, USA) and exposed in Molecular Imager ChemiDoc XRS System (Bio-Rad, Hercules, CA, USA).

### Cell Counting Kit-8 (CCK-8) Assay

Cells were seeded into 96-well plates (6.0×10^3^ cells/well). Cell viability was assessed by CCK-8 assay (Beyotime institute of biotechnology, China). The absorbance of each well was read on a spectrophotometer (Thermo, Pittsburgh, PA, USA) at 450 nm (A450). Three independent experiments were performed in quintuplicate.

### Flow Cytometric Analysis

Flow cytometric analysis was taken to detect the apoptosis and cell cycle status. The cells were harvested, washed twice, and resuspended in 100 µL of PBS containing 3 µL of annexin V and 3 µL of PI (KeyGen, China) according to the manufacturer’s recommendation. The apoptosis data acquisition and analysis were performed by the flow cytometry facility (BD Biosciences, San Jose, CA).

Cell cycle analysis was assessed by DNA content detected using the flow cytometry facility (BD Biosciences, San Jose, CA, USA). After collected by trypsinization, the cells were suspended with 70% ethanol and kept at 4°C for 30 min. Filtered through the mesh, and the DNA content of the stained nuclei was analysed. All experiments were performed three times to assure the results facticity.

### Tumorigenicity in Nude Mice

To investigate the effects of WT1 on tumor growth in vivo, we established the xenograft model in 4-week-old Balb/c^nu/nu^ mice. We employed 90 Balb/c^nu/nu^ mice (purchased from model animal research center of Nanjing University in China) in this study and divided them into 5 groups randomly (n = 6 per group) for each cell line (A549/H1299/H1650). All cells were tripsinized and resuspended with 100 µL of PBS (containing 50 µL Matrigel) respectively and subcutaneously injected into the axilla of each nude mouse (5×10^6^ cells per mouse). One week after injection, tumors dimensions were measured every 4 days and after one month all mice were sacrificed and tumors were obtained (Figure S2). The volume was calculated using following formula: volume = length×width^2^×0.5.

### Immunohistochemistry

Tissues were fixed in 4% paraformaldehyde and cut from paraffin block to 5 µm thickness. After dewaxing with xylene and rehydration with a graded series of ethanol, the slides were heated in the autoclave for three minutes using citrate buffer (PH 6.0) and incubated with primary antibody WT1(1∶100, 6F-H2, Millipore, USA), p-STAT3 (1∶400, Cell Signalling Technology, Beverly, MA, USA), Cyclin D1 (1∶50, Santa Cruz Biotechnology, Delaware Avenue, CA, USA) and p-pRb (1∶100,Cell Signaling Technology, Beverly, MA, USA) at 4°C overnight. Blocking serum or antibody dilution buffer were prepared as Negative controls. The primary antibodies utilized were all the same as for Western blot analysis. Photographs were taken by microcope (Nikon, ECLIPSE 50i) and software NIS-Elements v4.0. Average values of integrated optical density (IOD) were obtained from five random fields per slide by using Image-Pro Plus software (v5.0). Every data was detected three times at least.

### Statistical Analysis

Data was presented as mean±SD based on three separated experiments. The Student’s t-test, ANOVA and two-sided Fisher exact test was used to evaluate the statistical significance of differences in all pertinent experiments. A value of P<0.05 was considered as statistical significance, and P<0.001 was considered highly significant. All statistical analyses were analyzed using the SPSS program v17.0 (SPSS, Chicago, IL, USA).

## Results

### 1. WT1 mRNA Levels were Overexpressed in NSCLC Specimens

**Figure 1:**
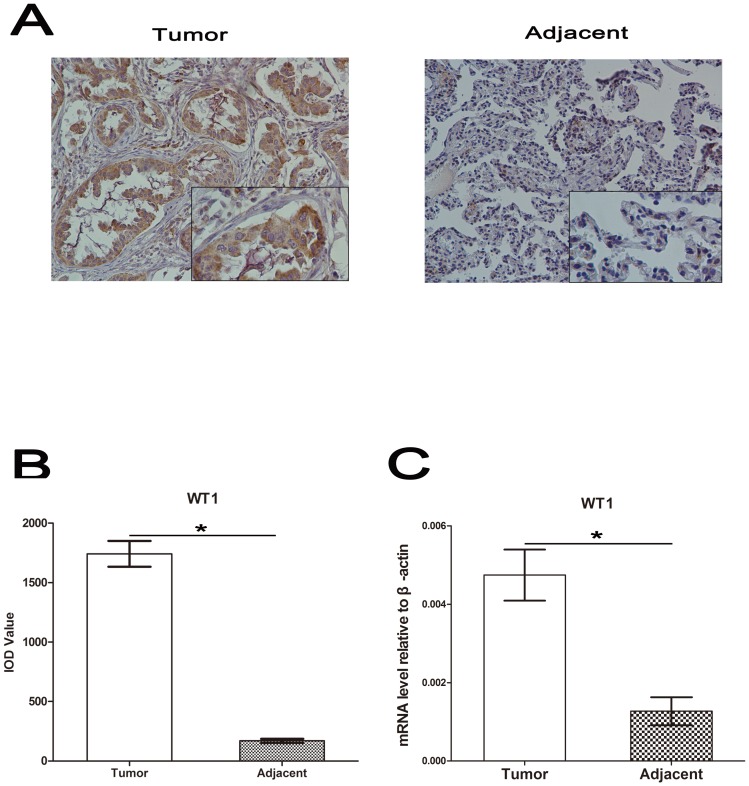
Up-regulation of WT1 in non-small cell lung cancer tissues. **A**, Immunohistochemical staining of WT1 in tumor (left) and adjacent (right) specimens. **B**, Average value of integrated optical density (IOD) was assessed by analyzing five fields per slide and recorded in the histogram. **C**, Real-time PCR analysis of WT1 mRNA level in tumor and adjacent tissues relative to β-actin. Data are represented as mean±SD. ^*^P<0.05, ^**^P<0.001.

We investigated 85 pairs of NSCLC and corresponding adjacent specimens using immunohistochemistry and real-time PCR. The clinical characteristics of the patients are shown in Table S1. WT1 expression in tumors was significantly higher compared to adjacent tissues. Representative pictures are shown in Figure 1A. Statistical analysis of IOD values of 85 slides stained with WT1 is shown in the histogram in Figure 1B (*p<0.05). As illustrated in Figure 1C, the mRNA level of WT1 was significantly overexpressed in NSCLC specimens (**p<0.001). Additionally, our findings indicated that WT1 mRNA level was associated both with tumor versus normal and with tumor stage, see Table S1(**p<0.001). Taken together, these results are in concordance with previous findings indicating that higher expression of WT1 is associated with NSCLC.

### 2. WT1 Expression Promoted the Viability of NSCLC Cell Lines in vitro

**Figure 2:**
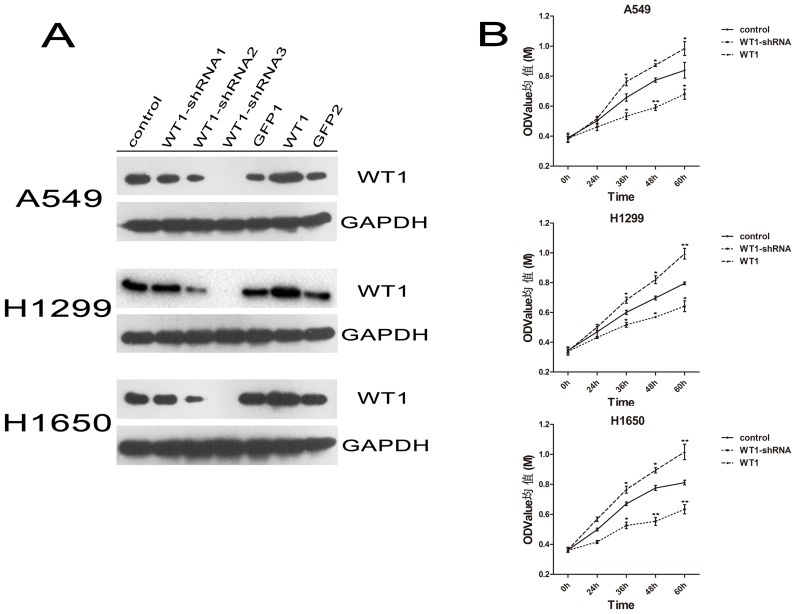
WT1 promotes NSCLC cell proliferation in vitro. **A** WT1 expression of NSCLC wild-type cells and NSCLC cells transfected by lentivirus containing pLL3.7 (GFP1), pLV-GFP (GFP2), pLL3.7-WT1-shRNA (WT1-shRNA1, WT1-shRNA2, WT1-shRNA3) and pLV-GFP-WT1 (WT1) by western-blot. **B**, The viability of NSCLC cells was assessed by CCK-8 assay: overexpression of WT1 promotes the cell viability while inhibition of WT1 expression reduces the effect. Data are represented as mean±SD. ^*^P<0.05, ^**^P<0.001.

To find a WT1-shRNA that could effectively down-regulate the expression of WT1 in NSCLC cell lines, three candidate WT1-shRNA (Table S2) were synthesized and tested using Western-blot analysis and real-time PCR. We found that WT1-shRNA3repressed the expression of WT1 in A549/H1299/H1650 with maximum efficiency (Figure 2A). Then, we detected the expression of WT1 in all cell lines to confirm the lentivirus by Western-blot analysis and real-time PCR. We found that the expression of WT1 in A549/H1299/H1650 transduced with pLL3.7-WT1-shRNA was much lower compared of the controls; simultaneously, the expression of WT1 in A549/H1299/H1650 transduced with pLV-GFP-WT1 was much higher compared to the controls. We also found that the two vectors (pLL3.7-WT1 and pLV-GFP) had no effect on the expression of WT1 (Figure 2A and Figure S3). Next, we conducted CCK-8 assay to test viability; the results indicated that overexpression of WT1 enhanced cell viability, whereas down-regulation of WT1 exhibited the opposite effect and the discrepancy was increasingly evident over time (Figure 2B). Therefore, these findings indicated that WT1 promoted NSCLC cell viability in vitro.

### 3. WT1 Expression Accelerated S-phase Entry of Cell Cycle by Up-regulating Cyclin D1 and p-pRb Protein

**Figure 3:**
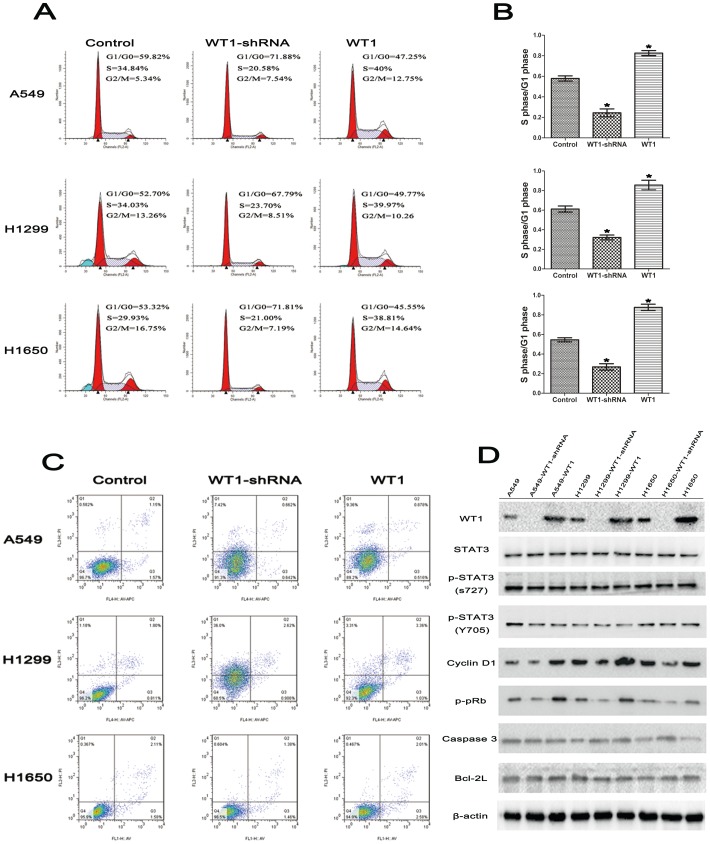
WT1 accelerates the S-phase entry of cell cycle by up-regulating Cyclin D1 and p-pRb. **A**, Cell cycle analysis of NSCLC wild-type cells and transduced with WT1-shRNA or WT1. Wild-type cells were used as control. **B**, The S-phase/G1-phase analysis was calculated and recorded in the histograms. **C**, Cell apoptosis analysis of NSCLC wild-type cells and those transduced with WT1-shRNA or WT1. Wild-type cells were used as control. **D**, Western-blotting analysis of expression of WT1, STAT3, p-STAT3 (S727 and Y705), Cyclin D1, p-pRb, caspase3, Bcl-2L in indicated NSCLC cells. GAPDH was used as a loading control. Data are represented as mean±SD. ^*^P<0.05, ^**^P<0.001.

To investigate the mechanism by which WT1 promoted NSCLC cell proliferation, we studied the effects of WT1 expression on the cell cycle via flow cytometric analysis. The results showed that the percentage of S-phase in WT1 overexpression group was higher compared to the control, whereas the WT1 knockdown group was lower (Figure 3A&3B). This result suggested that WT1 potentially promoted NSCLC cell proliferation by accelerating S-phase entry of cell cycle.

In order to further elucidate the mechanism, we detected the expression of Cyclin D1 and p-pRb because this activity is required for cell cycle G1/S transition by Western-blot. As illustrated in Figure 3D, Cyclin D1 and p-pRb protein were both increased in WT1 overexpressing cells and reduced in WT1 down-regulated cells. Based on WT1, enhanced transcriptional activity of p-STAT3, and other findings by Rong et al, we detected the activity of STAT3 and p-STAT3 (S727 and Y705) and found that phosphorylation of both S727 and Y705 was overexpressed in all cell lines. However, to date, there are no reports that have investigated whether WT1 is associated with the phosphorylation of STAT3. Additionally, we also detected apoptosis and did not find any effect of WT1 (Figure 3C&3D). Taken together, our findings suggest that WT1 accelerates S-phase entry of cell cycle by up-regulating Cyclin D1 and p-pRb protein expression.

### 4. WT1 Expression Promoted the Growth of Tumor Xenografts in vivo

**Figure 4:**
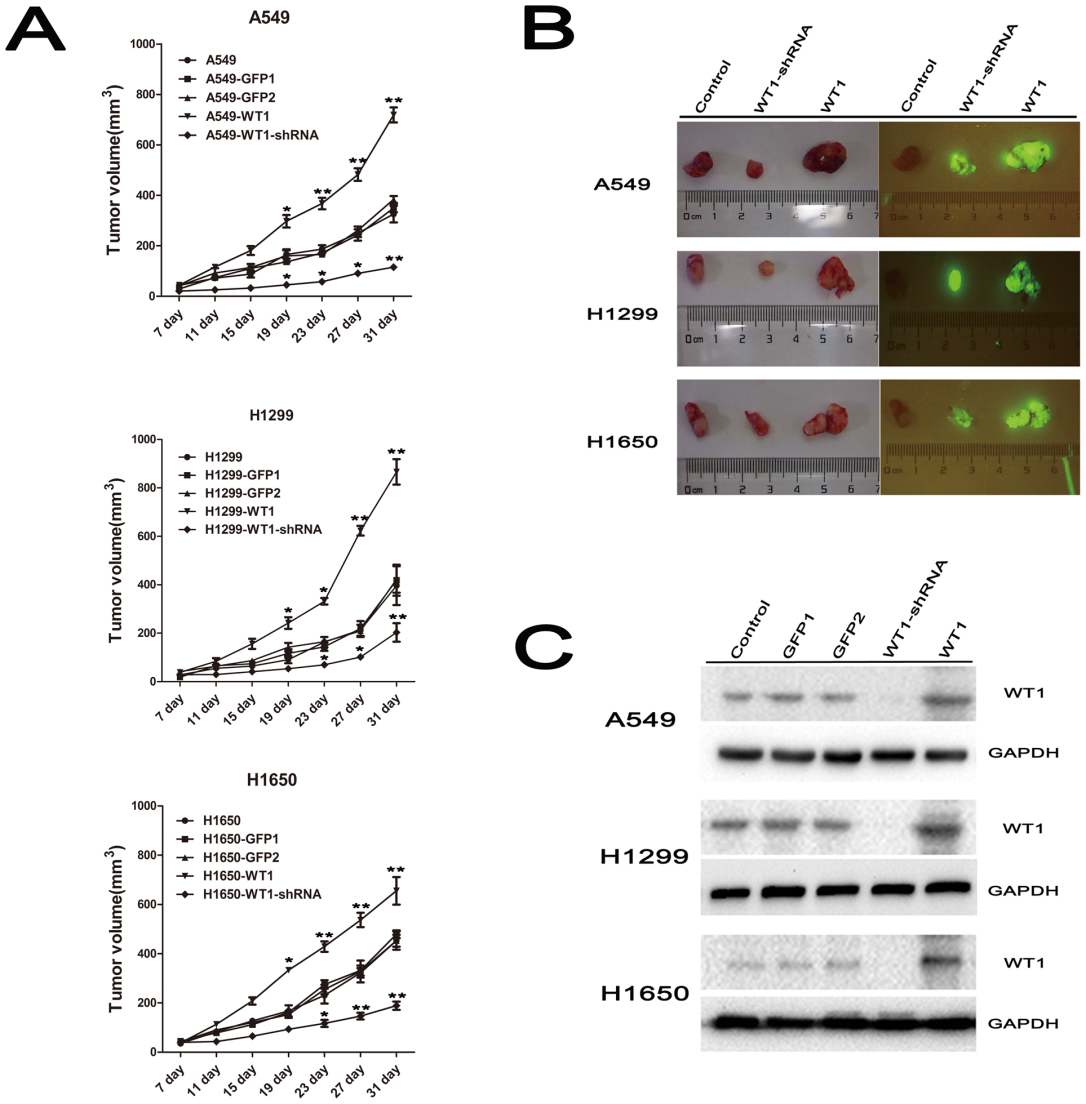
WT1 promotes the growth of tumor xenografts in vivo. **A**, Tumor volume of Balb/c^nu/nu^ nude mice 31 days after NSCLC cells injection. **B**, Tumor tissues obtained from nude mice presented in bright light (left) and in vivo fluorescence image system. **C**, WT1 expression in tumor tissues from nude mice by Western-blotting analysis. Data are represented as mean±SD. ^*^P<0.05, ^**^P<0.001.

To determine whether WT1 affected NSCLC progression in vivo, we established a xenograft tumor model using Balb/c^nu/nu^ mice. Mice were subcutaneously injected with stably pLV-GFP-WT1 and pLL3.7-WT1-shRNA infected A549/H1299/H1650 cells at a single site, respectively with empty plasmid and wild-type WT1 as controls. As illustrated in Figure 4A, we found that there were no significant differences between wild-type cells and cells transduced with pLV-GFP and pLL3.7-GFP; this indicated that the tumor growth curve in the cells transduced with pLV-GFP-WT1 was markedly increased compared with control, while the tumor growth curve in cells transduced with pLL3.7-WT1-shRNA was significantly inhibited. Tumor tissues obtained from nude mice were presented in bright light (Figure 4B left) and in vivo fluorescence image system (Figure 4B right). Significant increases in tumor volume were found when cells were transduced with pLV-GFP-WT1 compared to wild-type cells; in contrast, tumor volume of cells transduced with pLL3.7-WT1-shRNA was significantly decreased. Additionally, we detected the expression of WT1 protein in tumors obtained from nude mice by Western-blot analysis. We found that it there was no change compared to cells in vitro (Figure 2A right and Figure 4C). These results suggest that WT1 promotes growth of subcutaneous implant tumor in vivo.

### 5. WT1 Affected the Expression of Cyclin D1 and p-pRb in vivo

**Figure 5:**
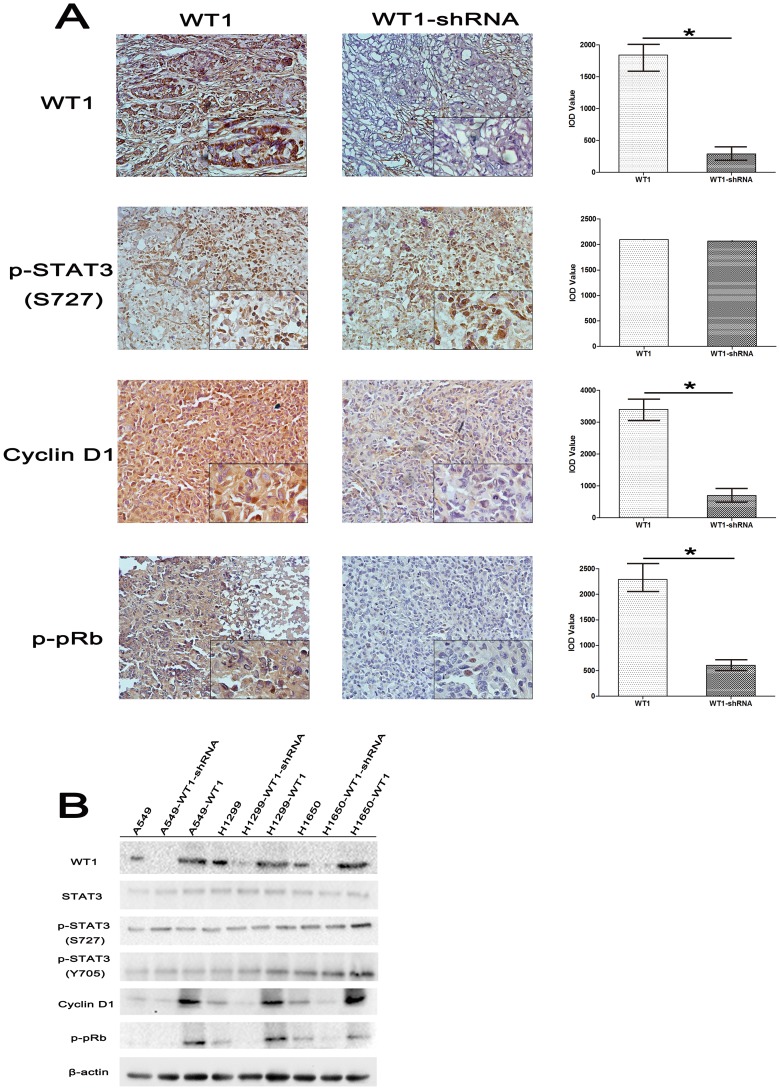
WT1 up-regulates the expression of Cyclin D1 and p-pRb in vivo. **A**, Immunohistochemical staining of WT1, p-STAT3 (s727), Cyclin D1 and p-pRb in WT1 overexpressed (WT1) tumor tissues and WT1 down-regulated (WT1-shRNA) tumor tissues in vivo. Average value of integrated optical density (IOD) was obtained as described above, demonstrated that the expression of Cyclin D1 and p-pRb was significantly up-regulated. **B**, Western-blotting analysis of expression of WT1, STAT3, p-STAT3 (S727 and Y705), Cyclin D1, p-pRb in indicated tumors. GAPDH was used as a loading control. Data are represented as mean±SD. ^*^P<0.05.

In vivo, we further validated our in vitro results in which WT1 accelerated S-phase entry of cell cycle by up-regulating Cyclin D1 and p-pRb. We investigated the expression of STAT3, p-STAT3 (S727), Cyclin D1 and p-pRb in tumors obtained from nude mice via immunohistochemical staining and Western-blot analysis. As shown in Figures 5A and 5B, the Cyclin D1 and p-pRb levels were increased in WT1 overexpressing tissues compared to WT1 down-regulated tissues. Meanwhile, p-STAT3 (S727) was overexpressed in both tissues. Statistical analysis of IOD values of tumor tissues is shown in the histogram (Figure 5A, p<0.05). Conclusively, these findings indicate that WT1 promotes growth of tumor in vivo and also depends upon up-regulation of the expression of Cyclin D1 and p-pRb.

### 6. WT1 Expression Affected the Expression of Cyclin D1 and p-pRb in NSCLC Specimens

**Figure 6:**
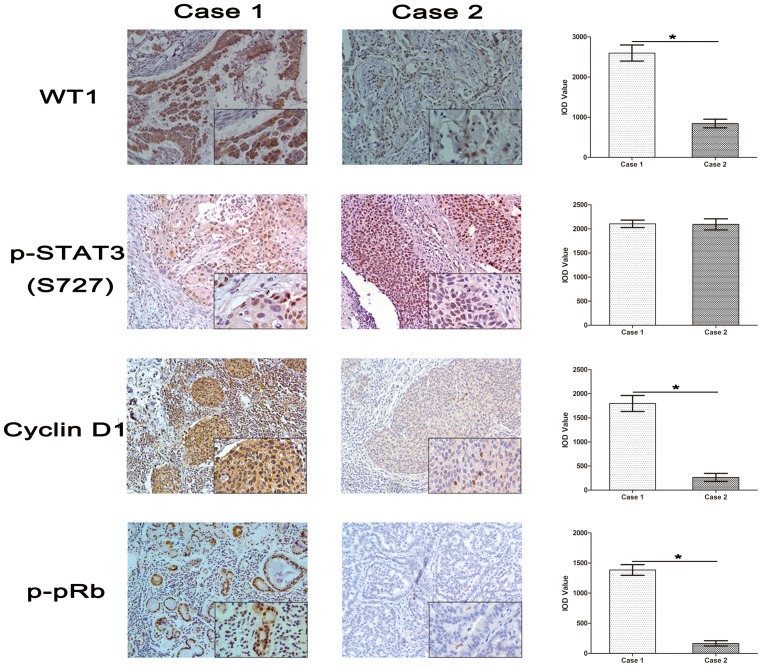
WT1 enhances the expression of Cyclin D1 and p-pRb in NSCLC specimens. Immunohistochemical staining of WT1, p-STAT3 (s727), Cyclin D1 and p-pRb in WT1 overexpression (Case 1) tumor tissues and WT1 low expression (Case 2) tumor tissues in vivo. Average value of integrated optical density (IOD) was obtained as described above, demonstrated that the expression of Cyclin D1 and p-pRb was significantly up-regulated. Data are represented as mean±SD. ^*^P<0.05.

We further evaluated the correlation between WT1 expression and the level of Cyclin D1 and p-pRb with 85 paraffin embedded human NSCLC tissue slides. Two cases with different WT1 expression levels are shown in Figure 6: Case1 (strong positive) and Case2 (weak positive). The level of Cyclin D1 and p-pRb was up-regulated in Case1 compared to Case2. As expected, p-STAT3 (S727) was strongly stained in both Case1 and Case2. This result supported the hypothesis that WT1 could increase the expression of Cyclin D1 and p-pRb and regulate the cell cycle.

## Discussion

Over the past several decades, although some studies have investigated the role of WT1 in NSCLC, its function has not been fully elucidated. In this study, we found that the expression of WT1 gene and protein in NSCLC specimens was markedly up-regulated compared with adjacent tissues; WT1 promoted proliferation of NSCLC cells in vitro and vivo, and WT1 expression affected the level of Cyclin D1 and p-pRb which accelerated cell proliferation in NSCLC cells and clinical specimens. With all findings taken together, we hypothesized that WT1 potentially plays an oncogenic role in promoting carcinogenesis and progression of NSCLC.

WT1 was originally identified as a tumor suppressor gene in Wilm’s tumor, and was subsequently found to be overexpressed in a variety of solid tumors [19]. However, the relationship between expression and carcinogenesis of WT1 in NSCLC remains controversial. Oji et al. suggested that WT1 might disturb the growth and differentiation of normal lung cells and contribute to oncogenesis of lung cancer [11]. More recently, Hayashi S et al reported that low WT1 gene expression in NSCLC tumors was a negative prognostic sign and was also associated with lymph node metastasis [20]. Moreover, it was demonstrated that WT1 loss induced senescence and decreased proliferation of lung cancer cells downstream of oncogenic KRAS signalling [21].STAT3 is constitutively activated in many human tumors such as prostate, lung, brain, breast and pancreatic cancer [13], [15], [22], [23]. The downstream genes including Cyclin D1, c-myc, and Bcl-xL of STAT3, are potentially up-regulated by phosphorylated STAT3. Cyclin D1 is a cell-cycle regulator that promotes cells from G1-phase to S-phase, and phosphorylates pRb protein [24], which is a nuclear phosphoprotein that regulates cell growth in G1-phase. Hypophosphorylated pRb on S780 then releases E2F from an inhibitory complex and enables it to promote the transcription necessary for progression into late G1-phase and S-phase [24]–[26]. It has been reported that Cyclin D1 and cyclin-depended-kinase 4 (CDK4) phosphorylated pRb and that pRb lost its ability to bind to E2F [27]. Thus, when Cyclin D1 is up-regulated by STAT3 it can phosphorylate pRb and promote cell growth by releasing E2F.

Rong et al has reported that the function of WT1 as tumor suppressor or oncogene was primarily dependent upon the activities of STAT3. Consequently when STAT3 was activated, WT1 functioned as a tumor suppressor, but when STAT3 was de-activated, WT1 functioned as an oncoprotein [18]. They also proposed that WT1 and STAT3 synergistically promoted cell proliferation by up-regulating genes such as Cyclin D1 and Bcl-xL. Based on these results, we hypothesized that WT1 could function as an oncogene in NSCLC.

In this study, we demonstrated that WT1 was overexpressed in NSCLC tissues compared with adjacent tissues. WT1 exhibited an effect on the proliferation of NSCLC cells in vitro and vivo: overexpression of WT1 promoted cell growth whereas down-regulation inhibited the proliferation of NSCLC cells. We also detected expression of STAT3 in NSCLC specimens and cells, in line with Fernandes et al who found STAT3 overexpressed in lung cancer tissues [23]. Our results showed that WT1 accelerated S-phase cell entry; thus, we assessed the cell cycle regulator genes such as Cyclin D1 and p-pRb and we found that the expression of Cyclin D1 and p-pRb was indeed up-regulated as shown in Figure 3D. Taking into consideration our results and the previous findings of Rong et al, we found that WT1 and STAT3 synergistically promote the growth of NSCLC cells by up-regulating the cell cycle regulators Cyclin D1 and p-pRb. Additionally, we found that WT1 expression was associated both with lymph node metastasis and tumor stage. This result indicates that WT1 expression may be relevant to tumor invasion and metastasis. Epithelial to mesenchymal transition (EMT) is considered an essential process for the metastasis of carcinoma and dissemination of cancer cells from the primary tumor and migration to different sites of the body [28]. Interestingly, WT1 could potentially drive EMT via EMT-related targets such as Snail, Slug and E-cadherin [29]–[31]. This will require further research to determine the function of WT1 in tumor invasion and metastasis.

Unexpectedly, we didn’t find that WT1 had any effect on the apoptosis of NSCLC cells according to flow cytometer assay and also by Western-blot assay; this is different from Rong Y et al’s findings that demonstrated WT1 increased the expression of Bcl-xL [18]. It was also reported that WT1 is required for inhibition of apoptosis in breast cancer and rhabdoid cancer by decreasing Bcl-2 mRNA and protein levels [32], [33]; however, other reports indicated that WT1 negatively regulated the Bcl-2 promoter in the prostate cell line [34]. Vincent S et al. reported that they did not detect any difference in response to WT1 depletion in NSCLC cell lines [21], which was in accordance with our findings. The reason for these differences remains unknown, but further elucidation, of why WT1 and STAT3 synergistically promote the level of Cyclin D1 but have no effect on the level of Bcl-2L in NSCLC, is warranted. The recently identified transcriptional WT1 co-factors, such as BASP1 (brain acid-soluble protein 1) and WTIP (WT1 interacting protein) might participate in these differences in WT1-mediated transcriptional regulation of target genes such as Bcl-2L [35]–[37].

In conclusion, in this study, we found a significantly higher WT1 expression level in NSCLC specimens compared to adjacent non-cancer tissues, we demonstrated the proliferation promoting function of WT1 in vitro and in vivo and we identified its oncogenic role in NSCLC via amplification of the transcriptional activity of p-STAT3 that up-regulates downstream genes, including Cyclin D1 and the hypo-phosphorylated retinoblastoma protein (p-pRb). Thus, WT1 potentially serve as a therapeutic target for the treatment of NSCLC.

## Supporting Information

Figure S1 The picture of NSCLC wild-type cells and others transfected with lentivirus in bright light (upper) and in green light (lower). NSCLC wild-type cells referred as control; cells transduced with pLL3.7 and pLV-GFP referred as GFP1 and GFP2; cells transduced with pLL3.7-WT1-shRNA referred as WT1-shRNA and transduced with pLV-GFP-WT1 referred as WT1 in the figure.
(TIF)

Figure S2 Tumors obtained from the nude mice. Tumors obtained from the nude mice are all presented in this figure. It should be noted that we only detected 4 tumors in H1299-WT1-shRNA group and 5 tumors in H1650-WT1-shRNA group in the injected site.
(TIF)

Figure S3 WT1 mRNA expression of NSCLC cells. WT1 expression of NSCLC wild-type cells and NSCLC cells transfected by lentivirus containing pLL3.7 (GFP1), pLV-GFP (GFP2), pLL3.7-WT1-shRNA (WT1-shRNA1, WT1-shRNA2, WT1-shRNA3) and pLV-GFP-WT1 (WT1) by Real-time PCR. Data are represented as mean±SD. *P<0.05.
(TIF)

Table S1 Relationship of WT1 expression and clinicopathological features of NSCLC.
(DOC)

Table S2 The sequence of WT1-shRNA.
(DOC)
