# Supplementary figures and images for: WT1 Promotes Cell Proliferation in Non-Small Cell Lung Cancer Cell Lines through Up-Regulating Cyclin D1 and p-pRb In Vitro and In Vivo
Source: PLoS One. 2013 Aug 1;8(8):e68837. doi: 10.1371/journal.pone.0068837 (PMC3731304; doi:10.1371/journal.pone.0068837)

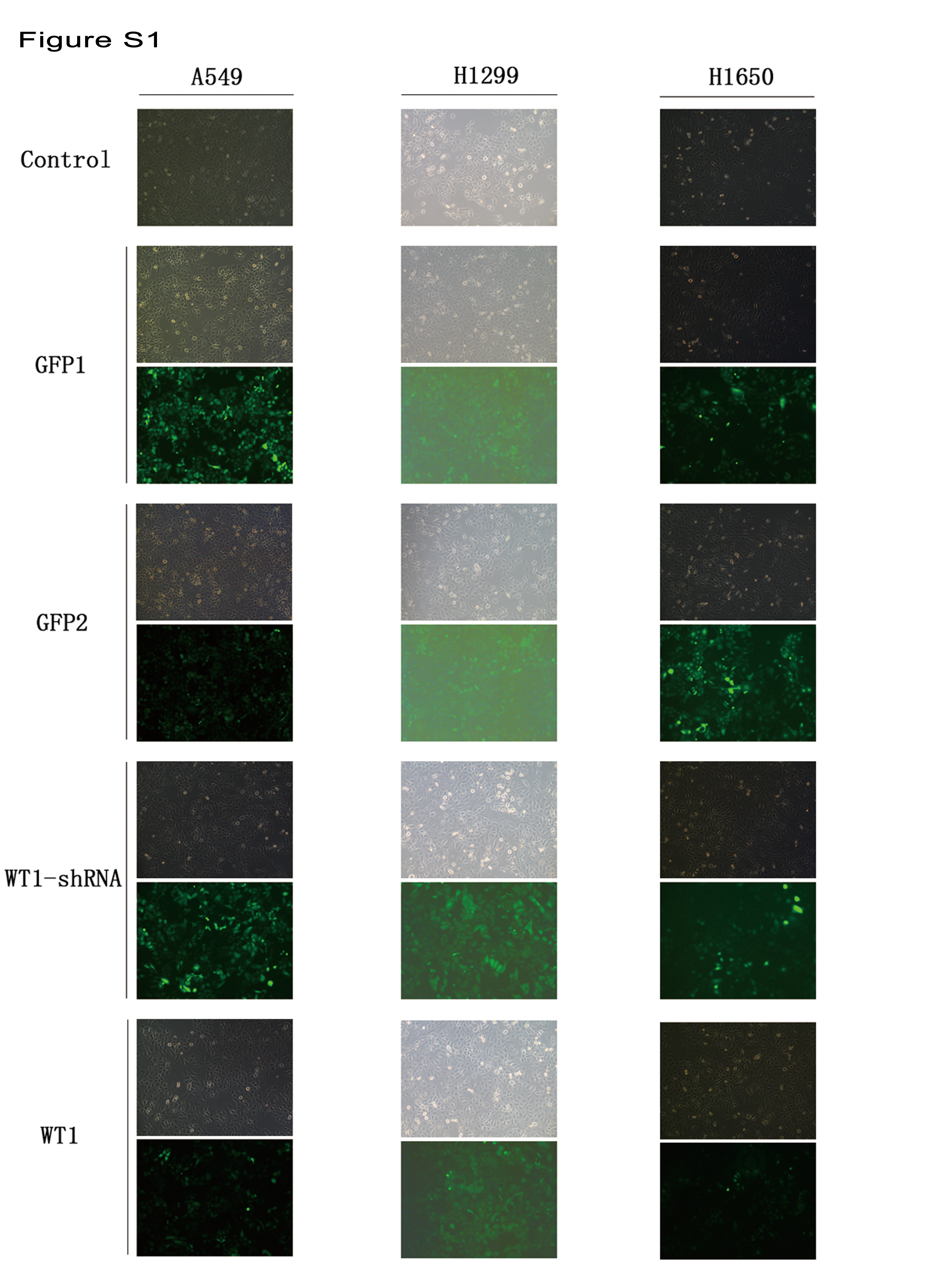

Supplement: Figure S1 — The picture of NSCLC wild-type cells and others transfected with lentivirus in bright light (upper) and in green light (lower). NSCLC wild-type cells referred as control; cells transduced with pLL3.7 and pLV-GFP referred as GFP1 and GFP2; cells transduced with pLL3.7-WT1-shRNA referred as WT1-shRNA and transduced with pLV-GFP-WT1 referred as WT1 in the figure. (TIF) [file pone.0068837.s001.tif]

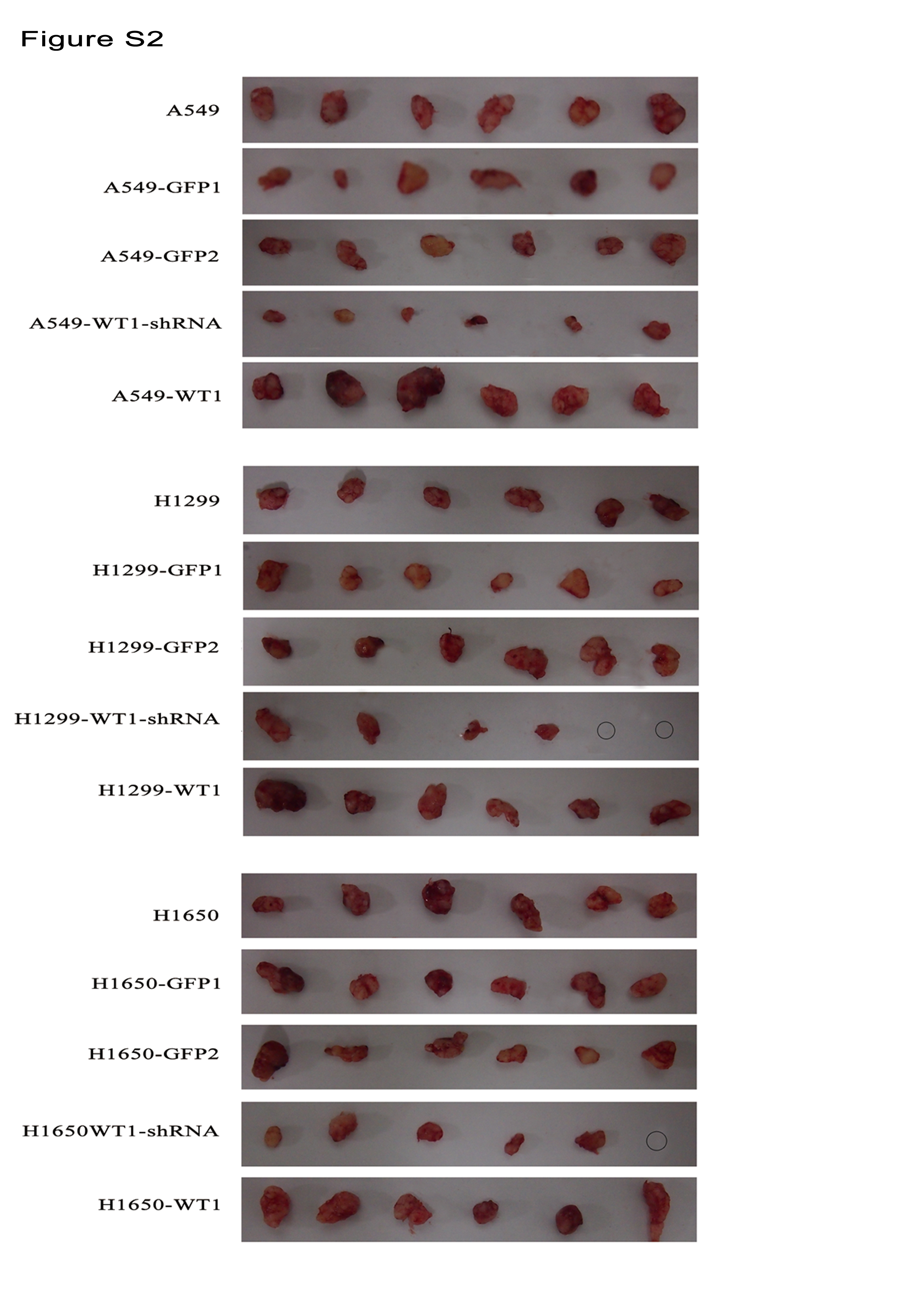

Supplement: Figure S2 — Tumors obtained from the nude mice. Tumors obtained from the nude mice are all presented in this figure. It should be noted that we only detected 4 tumors in H1299-WT1-shRNA group and 5 tumors in H1650-WT1-shRNA group in the injected site. (TIF) [file pone.0068837.s002.tif]
