# Supplementary material for: WT1 Promotes Cell Proliferation in Non-Small Cell Lung Cancer Cell Lines through Up-Regulating Cyclin D1 and p-pRb In Vitro and In Vivo
Source: PLoS One. 2013 Aug 1;8(8):e68837. doi: 10.1371/journal.pone.0068837 (PMC3731304; doi:10.1371/journal.pone.0068837)
Supplement: Table S1 — Relationship of WT1 expression and clinicopathological features of NSCLC. (DOC) [file pone.0068837.s004.doc]

**Table S1: Relationship of WT1 expression and clinicopathological features of NSCLC**

| **Clinicopathological**  **features** | **Sample**  **(n=85)** | **WT1 expression** | | | **X^2^** | **P** |
| --- | --- | --- | --- | --- | --- | --- |
|  |  | **Negative** | **Low** | **High** |  |  |
| **Gender** |  |  |  |  | 0.558 | 0.844 |
| Male | 47 | 3 | 16 | 28 |  |  |
| Female | 38 | 4 | 12 | 22 |  |  |
| **Age (years)** |  |  |  |  | 1.180 | 0.510 |
| ＜60  ≥60 | 39  46 | 2  5 | 16  12 | 21  29 |  |  |
| **Smoker** |  |  |  |  | 3.153 | 0.232 |
| No | 40 | 1 | 14 | 25 |  |  |
| Yes | 45 | 6 | 14 | 25 |  |  |
| **Histology** |  |  |  |  | 2.824 | 0.702 |
| Squamous carcinoma | 38 | 3 | 12 | 23 |  |  |
| Adenocarcinoma | 46 | 4 | 15 | 27 |  |  |
| Adenosquamous carcinoma  **Tumor**  T1/T2  T3/T4  **Lymph node metastasis**  N0  N1/N2/N3  **Distance metastasis**  M0  M1 | 1  56  29  38  47  85  0 | 0  4  3  7  0  7  0 | 1  15  13  15  13  28  0 | 0  37  13  16  34  50  0 | 3.698  13.036 | 0.170  0.001^**^ |
| **TNM Stage**  Early stage (Ⅰ‑Ⅱ) | 38 | 6 | 17 | 14 | 13.117 | 0.001^**^ |
| Advanced stage (Ⅲ‑Ⅳ) | 47 | 1 | 11 | 36 |  |  |

**IHC was scored according to the following criteria: +, 0~25% of the cells stained; ++, 26~50% of the cells stained; +++, 51~75% of the cells stained; ++++, 76%~100% of the cells stained. [score<+], [+ and ++] and [+++ and ++++] were represented as negative, low and high expression respectively.**

**^**p^＜0.05 was considered as statistically significant with the stage.**
