# Supplementary material for: WT1 Promotes Cell Proliferation in Non-Small Cell Lung Cancer Cell Lines through Up-Regulating Cyclin D1 and p-pRb In Vitro and In Vivo
Source: PLoS One. 2013 Aug 1;8(8):e68837. doi: 10.1371/journal.pone.0068837 (PMC3731304; doi:10.1371/journal.pone.0068837)
Supplement: Table S2 — The sequence of WT1-shRNA. (DOC) [file pone.0068837.s005.doc]

| Name | Sequence |
| --- | --- |
| WT1-shRNA1 | Forward:5'-aacCAAACTCCAGCTGGCGCTTTGAAttcaagagaTTCAAAGCGCCAG  CTGGAGTTTGttttttc-3'  Reverse:5'-tcgagAAAAAACAAACTCCAGCTGGCGCTTTGAATCTCTTGAATTC  AAAGCGCCAGCTGGAGTTTGgtt-3' |
| WT1-shRNA2 | Forward:5'-aacTAGTCCGCCATCACAACATGCATttcaagagaATGCATGTTGTGAT  GGCGGACTAttttttc-3'  Reverse:5'-tcgagAAAAAATAGTCCGCCATCACAACATGCATTCTCTTGAAATG  CATGTTGTGATGGCGGACTAgtt-3' |
| WT1-shRNA3 | Forward:5'-aacTCAGGGTTACAGCACGGTCttcaagagaGACCGTGCTGTAACCC  TGAttttttc-3'  Reverse:5'-tcgagaaaaaaTCAGGGTTACAGCACGGTCtctcttgaaGACCGTGCTGTA  ACCCTGAgtt-3' |

**Table S2: The sequence of WT1-shRNA**
